# Expanding the vicious cycle: the interplay between BPH, OSA, and circadian regulation in nocturia

**DOI:** 10.1038/s41440-025-02180-5

**Published:** 2025-03-18

**Authors:** Yu-Hsiang Lin, Kuo-Jen Lin, Chun-Te Wu

**Affiliations:** 1 Department of Urology, Chang Gung Memorial Hospital at Linkou, Taoyuan, Taiwan; 2 School of Medicine, Chang Gung University, Taoyuan, Taiwan

**Keywords:** Nocturia, ADH, BPH, OSA, ANP

**To the Editor**,

We read with great interest the recent article by Nagai et al. discussing the vicious cycle between nocturia and sleep blood pressure regulation [1]. Their hypothesis regarding atrial natriuretic peptide (ANP) and B-type natriuretic peptide (BNP) as central mediators of nocturia and nocturnal hypertension is insightful. However, we propose an expanded perspective that integrates both urological and sleep medicine viewpoints, emphasizing the roles of benign prostatic hyperplasia (BPH) and obstructive sleep apnea (OSA) in disrupting the suprachiasmatic nucleus (SCN) and its downstream effects on nocturnal blood pressure and urine production. Kato et al. demonstrated that nocturnal polyuria (NP) is significantly associated with attenuated nocturnal systolic blood pressure dipping [2], reinforcing Nagai et al.’s hypothesis that nocturia is not merely a urological symptom but also a cardiovascular concern. Similarly, Pinilla et al. confirmed that patients with OSA exhibit nondipping blood pressure patterns due to autonomic dysregulation [3]. These findings suggest that nocturia, hypertension, and OSA are interconnected beyond the cardiac volume overload hypothesis.

Our recent work highlighted that BPH contributes to nocturia not only via bladder outlet obstruction but also by influencing antidiuretic hormone (ADH) secretion [4]. Aging-related changes in SCN function may further exacerbate this dysregulation, impairing both ADH release and nocturnal blood pressure regulation. Similarly, OSA-related intermittent hypoxia and thoracic negative pressure surges have been implicated in ANP/BNP overactivation and ADH suppression, exacerbating nocturnal polyuria and hypertension [5]. We propose that BPH and OSA disrupt the SCN, leading to dysregulated ADH secretion, impaired blood pressure dipping, and nocturnal polyuria, forming a complex vicious cycle, as illustrated in Fig. 1, Proposed Pathophysiology of the Vicious Cycle in Nocturia and Hypertension. This model underscores the need for a multidisciplinary approach that integrates urology, sleep medicine, and circadian rhythm regulation in the management of nocturia-related cardiovascular morbidity.

**Fig. 1:**
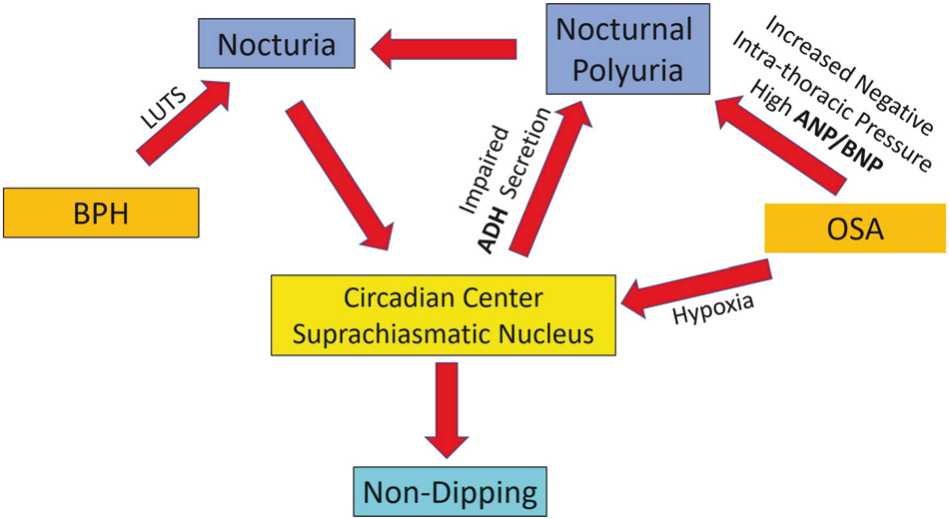
Proposed Pathophysiology of the Vicious Cycle in Nocturia and Hypertension. This schematic illustrates the interplay between benign prostatic hyperplasia (BPH), obstructive sleep apnea (OSA), and nocturnal blood pressure dysregulation, mediated through the suprachiasmatic nucleus (SCN). • BPH contributes to nocturia via lower urinary tract symptoms (LUTS), which leads to increased nighttime urination. • OSA induces increased negative intrathoracic pressure, triggering the excessive release of atrial natriuretic peptide (ANP) and B-type natriuretic peptide (BNP), leading to nocturnal polyuria. • OSA-induced hypoxia further disrupts the circadian regulation of the SCN, impairing antidiuretic hormone (ADH) secretion. • SCN dysfunction leads to impaired blood pressure dipping (“nondipping”), worsening nocturnal hypertension. • The vicious cycle formed between these mechanisms exacerbates nocturnal symptoms and cardiovascular burden. This model highlights the need for a multidisciplinary approach in the management of nocturia and nocturnal hypertension, incorporating insights from urology, sleep medicine, and circadian biology

While Nagai et al. provided an excellent foundation for understanding the nocturia-hypertension cycle, we suggest a broader framework incorporating BPH and OSA as key contributors to SCN dysfunction and ADH imbalance. Recognizing these mechanisms may lead to more effective interventions for nocturia and its associated cardiovascular burden.

## Data Availability

This Letter to the Editor is based on previously published literature and does not involve any new data collection by the authors. All analyses and discussions are derived from publicly available sources cited within the manuscript. Therefore, no datasets were generated or analyzed during this study.
